# The Emerging Fungal Pathogen *Candida auris* Induces IFNγ to Colonize the Skin

**DOI:** 10.1371/journal.ppat.1013114

**Published:** 2025-04-28

**Authors:** Diprasom Das, Shrihari M. Ganesh, Abtar Mishra, Mihai G. Netea, Shankar Thangamani

**Affiliations:** 1 Department of Comparative Pathobiology, College of Veterinary Medicine, Purdue University, West Lafayette, Indiana, United States of America; 2 Department of Internal Medicine and Radboud Center for Infectious Diseases, Radboud University Medical Center, Nijmegen, the Netherlands; 3 Department for Immunology and Metabolism, Life and Medical Sciences Institute (LIMES), University of Bonn, Bonn, Germany; 4 Purdue Institute for Immunology, Inflammation and Infectious Diseases (PI4D), West Lafayette, Indiana, United States of America; University of Michigan, UNITED STATES OF AMERICA

## Abstract

*Candida auris* is an emerging multidrug-resistant skin-tropic fungal pathogen that causes serious human infections. However, the factors that regulate *C. auris* skin infection *in vivo* are still unclear. In this study, we identified that, unlike *Candida albicans,* which induces IL-17-secreting protective effector Th17 cells, *C. auris* predominately induces IFNγ-secreting pathogenic Th1 cells during reinfection. Surprisingly, we found that IFNγ enhances skin infection of *C. auris* but not *C. albicans.* Mechanistically, IFNγ enhances skin infection of *C. auris* by dampening the protective IL-17 responses and increasing dermal damage. Furthermore, we identified that the development of Th1 cells occurs through IL-12, produced by *C. auris*-induced inflammatory macrophages and monocyte-derived dendritic cells. In addition, our findings reveal that *C. auris* unique cell wall outer mannan layer regulates the development of Th1 and Th17 cells. Collectively, our findings, for the first time, identified that *C. auris* induces IFNγ to persist in the skin. These findings help explain why *C. auris* but not *C. albicans* preferentially persist in the skin long-term, with the potential to identify novel therapeutic approaches to prevent and treat this emerging fungal pathogen in humans.

## Author summary

*C. auris*, an emerging fungal pathogen, preferentially colonizes the human and mouse skin. However, the host immune factors that regulate *C. auris* skin infection *in vivo* are poorly understood. This study identifies that *C. auris* predominately induces IFNγ-secreting pathogenic Th1 cells during reinfection. Excess IFNγ enhances skin infection of *C. auris* by dampening the protective IL-17 responses and increasing dermal damage. Furthermore, *C. auris* unique cell wall outer mannan layer regulates the development of Th1 cells in the skin. These findings, for the first time, identify the T cell immune responses to *C. auris* during reinfection that could open the door to developing novel vaccine strategies to prevent and treat this fungal pathogen.

## Introduction

*C. auris* was recently classified within the critical priority fungal pathogens group by the World Health Organization (WHO) and categorized as an urgent threat by the US Centers for Disease Control and Prevention (CDC) [1–5]. Unlike other *Candida* species, such as *Candida albicans* that colonizes the intestinal tract, *C. auris* preferentially colonizes the human skin, leading to nosocomial transmission and outbreaks of systemic fungal infections [6–8]. Furthermore, unlike skin-tropic fungal pathogens such as *Malassezia* [9], *C. auris* not only colonizes the epidermis of the skin but also enters the deeper dermis, a phenomenon that was not observed previously [6]. *C. auris* can persist in skin tissues for several months and evade routine clinical surveillance [6,10]. Given that most *C. auris* isolates exhibit resistance to several FDA-approved antifungal drugs, a deeper understanding of the antifungal host defense mechanisms is critical to developing new host-directed therapeutic approaches to prevent and treat this newly emerging skin tropic fungal pathogen. Because skin infection is a prerequisite for *C. auris* transmission and subsequent invasive disease, understanding the immune factors involved in local skin defense against *C. auris* is important to prevent invasive infection.

The fungal cell wall components represent the predominant pathogen-associated molecular patterns (PAMPs) directly interacting with the host to orchestrate the antifungal immune response [11]. Recent evidence indicates that the cell wall of *C. auris* is structurally and biologically unique compared to other *Candida* species, including *C. albicans* [12]. The outer cell wall mannan layer in *C. auris* is highly enriched in β-1,2-linkages and contains two unique Mα1-phosphate side chains not found in other *Candida* species [12]. *C. auris* differentially stimulates cytokine production in peripheral blood mononuclear cells, decreases phagocytosis by neutrophils, and has a more potent binding to IgG than *C. albicans* [13–15]. Using a single-cell RNA sequencing approach, we recently identified the cell type-specific skin immune responses to *C. auris* infection [16]. However, almost nothing is known regarding the immune responses against *C. auris* during reinfection. The potential role of T cell responses in establishing resistance to secondary infection of *C. auris* in the skin remains unknown, and as a result, developing effective fungal vaccines remains an elusive goal [17]. Therefore, understanding the T cell immune responses during reinfection in the skin is important to developing novel preventive approaches, including vaccine strategies, to prevent and treat this emerging fungal pathogen in humans. Because *C. auris* possesses a unique outer cell wall layer and preferentially colonizes and persists in skin tissue long-term, effector T cell responses from other fungal pathogens, including *C. albicans*, cannot be extrapolated to *C. auris* during reinfection. Therefore, this study focused on defining the role of effector T cell responses during *C. auris* reinfection in the skin.

In this study, we show that unlike *C. albicans,* which induces Th17 cells, *C. auris* predominately induces Th1 cells in addition to Th17 cells. Surprisingly, we found that the Th1 cytokine IFNγ enhances skin infection by *C. auris* but not *C. albicans.* Our results show that the development of Th1 cells occurs through IL-12, produced by *C. auris*-activated macrophages and monocyte-derived dendritic cells. Furthermore, our findings reveal that *C. auris* unique cell wall outer mannan layer regulates the development of Th1 and Th17 cells. Collectively, we have identified the T cell responses during *C. auris* reinfection and identified pathways divergent from *C. albicans.* These findings help explain why *C. auris* but not *C. albicans* preferentially colonizes the skin long-term, with the potential to identify novel therapeutic approaches to prevent and treat this emerging fungal pathogen in humans.

## Results

### *C. albicans* induces Th17 cells, whereas *C. auris* predominately induces interferon-γ (IFN-γ)–producing Th1 cells in the skin

**Fig 1:**
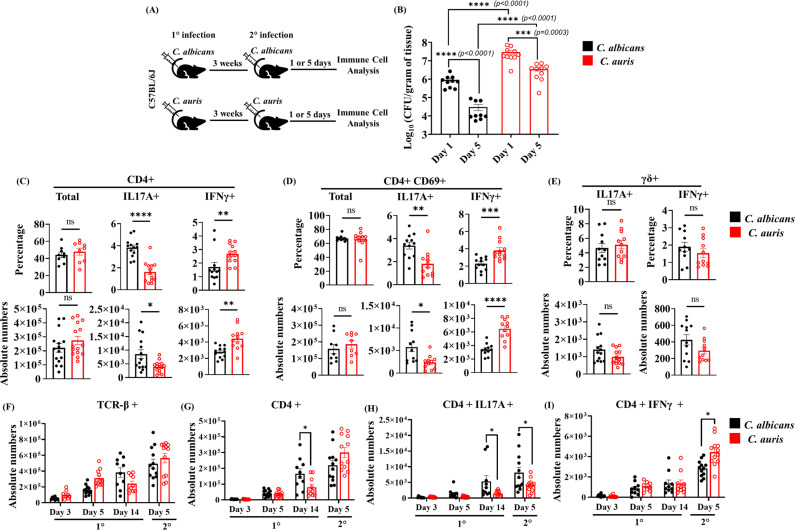
*C. albicans* induces Th17 cells, whereas *C. auris* predominately induces interferon-γ (IFN-γ)–producing Th1 cells in the skin. **(A)** C57BL/6J mice were intradermally infected with either *C. albicans* or *C. auris* (dose ~ 1–2 x 10^6^/ mice) on the dorsal skin and allowed to rest for 3 weeks. Mice were re-infected with the respective *C. albicans* or *C. auris* strains. After 5 days of post-secondary infection, fungal burdens and immune cells were analyzed in the infected skin portion. **(B)** Skin fungal burden in the infected *C. albicans* or *C. auris* infected group of mice after 1- and 5-day post-secondary infection (n = 9–10 mice per group). **(C)** Percentage and absolute cell numbers (per gram tissue) of total CD4+ (gated from TCR- β+), CD4 + IL17A + , and CD4 + IFNγ+ (gated from TCR-β+ CD4+) T cells in the infected skin tissue (n = 9–14 mice per group). **(D)** Percentage and absolute cell numbers (per gram tissue) of total CD4 + CD69+ (gated from CD4 + CD44 + cells), CD4 + CD69 + IL17A + , and CD4 + CD69 + IFNγ+ (gated from CD4 + CD44 + CD69 + cells) T cells in the infected skin tissue (n = 9–12 mice/group). **(E)** Percentage and absolute cell numbers (per gram tissue) of total γδ + IL17A + and γδ + IFNγ+ (gated from γδ+ cells) T cells in the infected skin tissue (n = 9–13 mice/group). **(F-I)** The absolute cell numbers (per gram tissue) of TCR-β+, CD4 + , CD4 + IL17A + , and CD4 + IFNγ+ cells in the indicated time points in *C. albicans* or *C. auris* infected mice post-primary or post-secondary infection. 1° represents primary infection and 2° represent secondary infection. Error bars represent mean ± SEM. ns - non-significant, * p < 0.05, ** p < 0.01, *** p < 0.001, **** p < 0.0001. Statistical significances were calculated using the Mann-Whitney *U* test. The black bar represents “*C. albicans* infected groups,” and the red bar represents “*C. auris* infected groups”.

To understand the effector T cell responses during *C. auris* skin reinfection, we infected mice intradermally with *C. auris* and rested them for 3 weeks. Mice were then reinfected with *C. auris*, and after 1 or 5 days of post-secondary infection, skin tissues were collected to determine the fungal load and T-cell responses. Another group of mice that received primary and secondary infection with *C. albicans* was used as a control to compare the fungal load and T cell immune responses with *C. auris,* as outlined in Fig 1A. We observed a significantly higher fungal load in the skin of the *C. auris*-infected mice when compared to the *C. albicans*-infected group on 1 and 5 days post-secondary infection (Fig 1B). Furthermore, fungal burden from day 1 to day 5 post-infection in *C. albicans*-infected mice showed almost 1.48 ± 0.13 log decrease, whereas *C. auris*-infected mice decreased only 0.92 ± 0.13 log. Next, we examined the development of Th1 and Th17 responses during reinfection. We identified that the percentage and absolute number of total CD4 + IL-17A + T cells and activated CD4 + CD69 + IL-17A + T cells were significantly decreased, whereas total CD4 + IFNγ+ T cells and activated CD4 + CD69 + IFNγ+ T cells were significantly increased in the skin of the *C. auris*-infected mice compared to the *C. albicans*-infected group (Figs 1C, 1D, S1A and S1B). Previous findings from our laboratory suggest that γδ + T cells also induce IL-17A and IFNγ expression during *C. auris* primary infection [16]. However, we identified no significant differences in γδ + IL-17A + and γδ + IFNγ+ T cells between *C. auris* and *C. albicans* groups during reinfection (Fig 1E). These results suggest that *C. albicans* cutaneous infection induces predominantly Th17 cells, whereas *C. auris* induces Th1 responses in the skin during reinfection.

Next, we aimed to understand if Th1 and Th17 responses differ between *C. auris* and *C. albicans* infection during primary and secondary infection. We examined the kinetics of total TCRβ+ T cells, CD4 + T cells, CD4 + IL17A+ cells, and CD4 + IFNγ+ cells in the skin tissue of mice that were infected once (primary infection) or reinfected (secondary infection) with *C. albicans* or *C. auris.* After primary infection, the number of TCRβ+ T cells was not significantly different between *C. auris* and *C. albicans*-infected groups on 3-, 5-, and 14 days post-infection (Fig 1F-1I). On day 14 after primary infection, the total CD4 + T cells and CD4 + IL17A+ cells were significantly decreased in the *C. auris*-infected groups compared to *C. albicans*-infected groups. In addition, CD4 + IFNγ+ cells were not significantly altered 3, 5, and 14 days after primary infection in the *C. auris* and *C. albicans* infection (Fig 1F-1I). After secondary infection, total TCRβ+ T cells and CD4 + T cells were not significantly different between *C. auris* and *C. albicans* infected groups. However, secondary infection significantly decreased the absolute number of CD4 + IL17A + T cells in *C. auris*-infected mice. On the other hand, CD4 + IFNγ+ T cells were significantly increased in the *C. auris*-infected groups compared to *C. albicans* infection (Fig 1F-1I). Collectively, our results show that *C. auris*-infected mice had a significantly higher fungal load upon re-infection than *C. albicans*-infected mice. Furthermore, the increased fungal load in the *C. auris* groups correlates with the potent Th1 response observed after secondary infection.

### IFNγ enhances the fungal burden of *C. auris* by dampening the protective IL-17 responses and increasing dermal damage.

**Fig 2:**
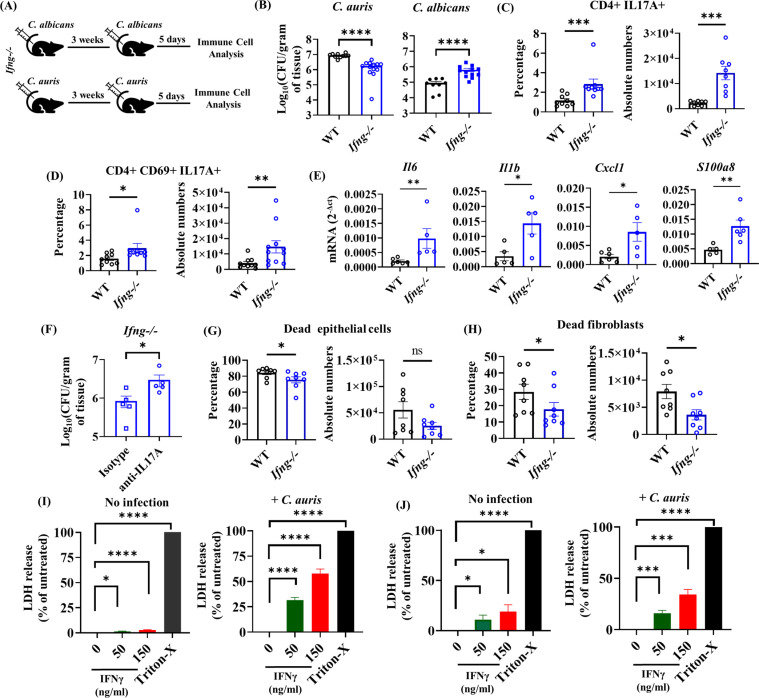
IFN γ **enhances skin infection of *C. auris* but not *C. albicans* by dampening the protective IL-17 responses and increasing dermal damage.** (A) *Ifng-/-* mice were intradermally infected with *C. albicans* (or) *C. auris*. After 3 weeks of primary infection, mice were re-infected with the respective *Candida* strains. After 5 days of post-secondary infection, fungal burdens and immune cells were analyzed in the infected skin tissue. (B) Skin fungal burden in the *C. auris* or *C. albicans*-infected WT and *Ifng-/-* mice after 5 days post-secondary infection (n = 9-10 mice per group). (C) Percentage and absolute cell numbers (per gram tissue) of CD4 + IL17A+ (gated from TCR-β+ CD4 + cells) T cells in the *C. auris*-infected WT and *Ifng-/-* mice (n = 9-10 mice per group). (D) Percentage and absolute cell numbers (per gram tissue) of CD4 + CD69 + IL17A+ (gated from CD4 + CD44 + CD69 + cells) T cells in the *C. auris*-infected WT and *Ifng-/-* mice (n = 9-10 mice per group). **(E)** mRNA analysis of *Il6, Il1b, Cxcl1* and *S100a8* in the skin of *C. auris*-infected WT and *Ifng-/-* mice after 5 days post-secondary infection (n = 5-6 mice/group). (F) Skin fungal burden in the *C. auris*-infected *Ifng-/-* mice determined after 5 days post-secondary infection that receives anti-IL17A or isotype antibody (n = 5 mice per group). (G-H) Percentage and absolute cell number (per gram tissue) of dead CD45^-^CD31^-^Tie2^-^EpCAM^+^ epithelial cells and dead CD45^-^ CD31^-^ Tie2^-^ EpCAM^-^ PDGFRα^+^ fibroblasts in the *C. auris*-infected WT and *Ifng-/-* mice determined 5 days post-secondary infection (n = 8 mice per group). (I) Lactate dehydrogenase (LDH) release was assessed after 24 hours of *C. auris* infection or no infection in mouse primary keratinocytes isolated from WT mice and exposed to indicated concentrations of IFNγ (ng/ml) (combined data from three independent experiments). Triton-X was used as a positive control. (J) LDH release was assessed after 24 hours of *C. auris* infection or no infection in mouse primary fibroblasts isolated from WT mice and exposed to indicated concentrations of IFNγ (ng/ml) (combined data from three independent experiments). Triton-X was used as a positive control. Error bars represent mean ± SEM. ns - non-significant, * p < 0.05, **p < 0.01, *** p < 0.001, **** p < 0.0001. For mice studies, statistical significances were calculated using the Mann-Whitney *U* test. For *ex-vivo* LDH assays, statistical significances were calculated using the paired t-test.

Th1 cells protect the host against fungal pathogens, including *C. albicans* [18,19]. However, despite inducing potent Th1 responses, *C. auris*-infected groups had significantly increased fungal load in skin tissues compared to *C. albicans-*infected groups. Therefore, we examined if IFNγ regulates fungal burden during *C. auris* or *C. albicans* skin infection. Wild type (WT) and *Ifng-/-* mice were intradermally infected with *C. auris* or *C. albicans* and rested for 3 weeks. Each group was re-infected with the respective fungal strains, and the fungal load was determined five days after infection, as outlined in Fig 2A. Surprisingly, we found that the skin tissue of *Ifng-/-* mice infected with *C. auris* had significantly decreased fungal load compared to WT groups. On the other hand, we observed a significantly increased fungal load in the skin tissue of *Ifng-/-* mice compared to WT mice infected with *C. albicans* (Fig 2B). Since both CD4 + and CD8 + T cells induce IFNγ+ during *C. auris* primary infection [16], we examined the contribution of CD8 + T cells in regulating *C. auris* during reinfection. After 3 weeks of primary infection, wild-type mice were administered anti-CD8 intraperitoneally on days -2 and + 2 during secondary *C. auris* skin infection. Fungal burden in the skin tissues was determined on day 5 of post-secondary infection and compared with mice that received no antibody. We identified that CD8 + T cell depletion did not significantly alter the skin fungal burden relative to control groups (S1C Fig). These results suggest that IFNγ produced by CD4 + T cells but not CD8 + T cells potentially contribute to increased fungal burden during *C. auris* skin reinfection. Our findings, along with others, suggest that Th1 cytokine IFNγ is critical to protecting the host against *C. albicans* [18,19]. On the other hand, we identified that IFNγ enhances fungal burden during *C. auris* infection. Since IFNγ and IL-17 can cross-regulate each other [20] and IL-17 is critical for host defense against *C. auris* [6], we hypothesized if increased IFNγ diminishes protective IL-17 response and increases fungal burden during *C. auris*-infection. Therefore, we examined the Th17 response in the *Ifng-/-* and WT mice infected with *C. auris.* We identified that the percentage and absolute number of total CD4 + IL17A+ cells and activated CD4 + CD69 + IL17A+ cells were significantly increased in the *Ifng-/-* mice compared with WT mice infected with *C. auris* (Fig 2C and 2D).

To understand if IFNγ dampens protective IL-17 response during secondary infection with *C. auris*, we examined the transcripts associated with IL-17 signatures, such as *Il6, Il1b, Cxcl1*, and *S100a8.* We found that *Il6, Il1b, Cxcl1*, and *S100a8* transcripts associated with IL-17 signature were significantly increased in the *Ifng-/-* mice compared with WT mice infected with *C. auris* (Fig 2E). Further to confirm IFNγ dampens protective IL-17 response and IL-17 is necessary to protect the host from secondary infection against *C. auris*, we infected *Ifng-/-* mice with *C. auris* and treated with IL-17 neutralization or isotype antibody on -2, 0 and + 2 days during secondary infection. We identified that *Ifng-/-* mice that received anti-IL-17 neutralization antibody showed significantly increased fungal burden compared to *Ifng-/-* mice that received isotype antibody (Fig 2F). These findings confirm that increased IFNγ dampens protective IL-17 response and IL-17 is necessary to protect the host from secondary infection against *C. auris.*

Since IFN-γ induces apoptosis in epithelial cells and fibroblasts and can enhance cell-mediated cytotoxicity [21–23], we examined if the increased production of IFN-γ in *C. auris*-infected mice might promote increased cell death and enhance fungal burden. We examined the cell death in CD45^-^CD31^-^Tie2^-^ EpCAM^+^ epithelial cells and CD45^-^ CD31^-^ Tie2^-^ EpCAM^-^PDGFRα+ fibroblasts in the skin tissue of *C. auris*-infected WT and *Ifng-/-* mice after 5 days post-secondary infection (S2A Fig). We observed that dead epithelial cells and fibroblasts were significantly increased in *C. auris*-infected WT mice compared to *Ifng-/-* mice (Fig 2G and 2H). Based on these findings, we examined if IFNγ directly causes cell death in skin keratinocytes and fibroblasts. We found that keratinocytes and fibroblasts isolated from WT mice and treated with IFNγ *in vitro* caused dose-dependent cell death, which was measured by lactate dehydrogenase (LDH) release. *C. auris* infection further enhances the IFNγ-induced cell death in keratinocytes and fibroblasts *in vitro* (Fig 2I and 2J). In addition, we identified that IFNγ treated keratinocytes and fibroblasts have significantly increased fungal growth compared to non-treated groups (S2B and S2C Fig). These findings suggest that excess IFNγ directly promotes dermal damage, leading to *C. auris* infection. Taken together, our findings suggest that IFNγ enhances skin infection of *C. auris* by dampening the protective IL-17 responses and increasing dermal damage.

### The development of Th1 cells during reinfection occurs through IL-12 produced by *C. auris*-induced inflammatory macrophages and monocyte-derived dendritic cells.

**Fig 3:**
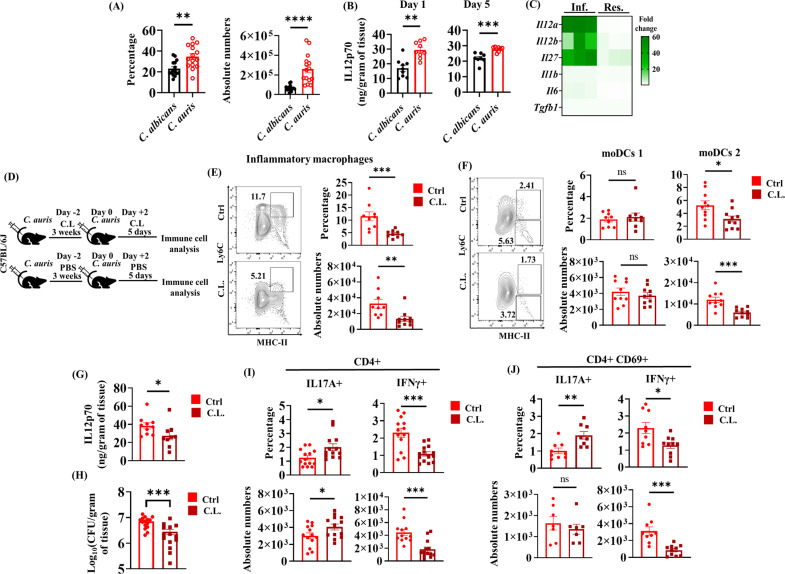
The development of Th1 cells occurs through IL-12 produced by *C. auris*-induced inflammatory macrophages and monocyte-derived dendritic cells. (A) Percentage and absolute cell numbers (per gram tissue) of total macrophages in the *C. albicans* or *C. auris*-infected skin after 5 days of post-secondary infection. (n = 9-12 mice per group). (B) Quantification of IL12(p70) level in the skin homogenate of *C. albicans* or *C. auris*-infected WT mice at 1- and 5-day post-secondary infection (n = 8-10 mice per group). (C) Heat map represents the relative fold change of normalized gene counts in the skin tissue of *C. auris-*infected samples relative to the uninfected group in inflammatory (Inf.) and resident (Res.) macrophages determined by scRNA-Seq 12 days after primary infection. (D) C57BL/6J mice were intradermally infected with *C. auris* 0387 and allowed to rest for 3 weeks. On day -2 and + 2 of re-infection with *C. auris,* mice were treated with 1mg of clodrosome (C.L.) (or) 1X PBS (Ctrl) as outlined. After 5 days of post-secondary infection, fungal burden, cytokine level, and immune cells were determined. (E-F) Representative flow plots, percentage, and absolute cell numbers (per gram tissue) of inflammatory (CD11b + CD64 + Ly6C^high^MHC-II+) macrophages (gated from CD11b+Ly6G-CD64 + cells), monocyte-derived dendritic cells 1 (moDC 1), and monocyte-derived dendritic cells 2 (moDC 2) gated from CD11b + Ly6G-CD64- cells in the skin tissue of control (Ctrl) and macrophage-depleted (C.L.) mice re-infected with *C. auris* (n = 8-10 mice/group). (G-H) IL12(p70) level and fungal burden in the skin tissue of Ctrl and C.L. groups (n = 9-13 mice/group). (I) Percentage and absolute cell number (per gram) tissue of CD4 + IL17A + and CD4 + IFNγ+ (gated from TCR-β+ CD4 + cells) T cells in the skin tissue of Ctrl and C.L. groups (n = 11-15 mice/group). (J) CD4 + CD69 + IL17A + and CD4 + CD69 + IFNγ+ T cells (gated from CD4 + CD44 + CD69 + cells) in the skin tissue of Ctrl and C.L. groups (n = 7-10 mice/group). Error bars represent mean ± SEM. ns – non-significant, * p < 0.05, ** p < 0.01, *** p < 0.001, **** p < 0.0001. Statistical significances were calculated using the Mann-Whitney *U* test.

Next, we aimed to understand the factors that regulate the development of Th1 cells during secondary infection of *C. auris*. IL-12 is critical for developing Th1 cells, and IL-12 produced by macrophages can modulate the Th1 immune response during re-infection with *Listeria monocytogenes* [24]. Therefore, we examined the macrophage and IL-12 levels in the skin tissue of *C. auris-*infected mice and compared them with *C. albicans*-infected groups. We found that after secondary infection, the percentage and absolute number of macrophages were significantly increased in the *C. auris*-infected mice than in *C. albicans*-infected skin tissue (Fig 3A). Similarly, compared to *C. albicans*-infection, IL-12p70 level was significantly increased in the skin tissue of *C. auris*-infected skin tissue on 1 and 5 days post-secondary infection (Fig 3B). Furthermore, using a single-cell RNA sequencing approach, we examined the expression of cytokines in macrophages during *C. auris* skin infection *in vivo*, which is critical for Th1 and Th17 development. We identified that Th1 polarizing cytokines such as IL-12 and IL-27 expressions were considerably increased in Ly6C^high^ inflammatory macrophages but not Ly6C^low^ skin resident macrophages. On the other hand, Th17 cytokines such as IL-1b, Tgfb1, and IL-6 were not considerably altered in the *C. auris*-infected mice compared to uninfected groups (Fig 3C). These results indicate that *C. auris* infection induces Th1 polarizing cytokines, including IL-12, in macrophages that could potentially regulate the development of Th1 cells after secondary infection.

**Fig 4:**
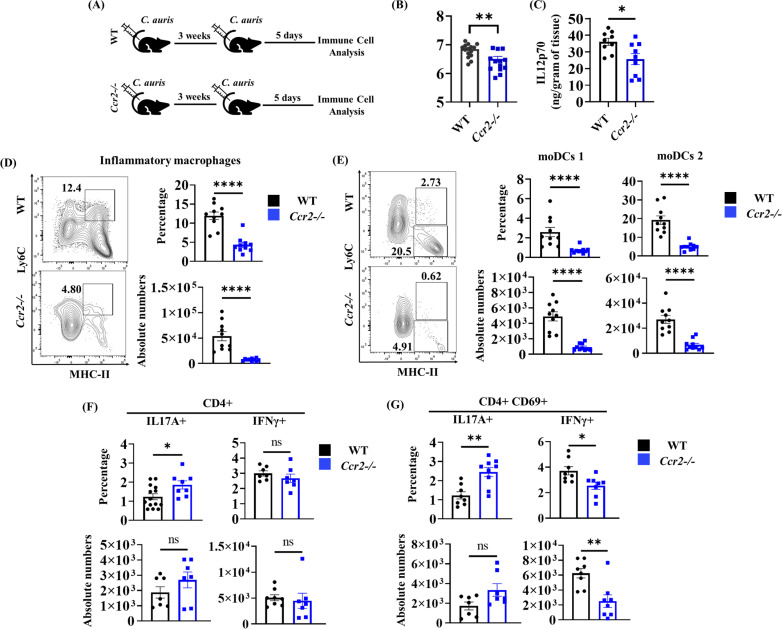
Th1 and Th17 cells in *Ccr2-/-* mice during reinfection with *C. auris.* (A) WT and *Ccr2-/-* mice were intradermally infected with *C. auris* 0387 and allowed to rest for 3 weeks. Mice were reinfected with the same dose of *C. auris* 0387, and after 5 days of reinfection, fungal burdens and immune cells were analyzed in the infected skin tissue. (B-C) Skin fungal burden and IL12(p70) level in the skin tissue of WT and *Ccr2-/-* mice at 5 days of post-secondary infection (n = 9-13 mice/group). (D-E) Representative flow plots, percentage, and absolute cell numbers (per gram tissue) of inflammatory (Ly6C ^high^ MHC-II+) macrophages (gated from CD11b + Ly6G- CD64 + cells), moDCs 1, and moDCs 2 (gated from CD11b + Ly6G-CD64- cells) in the skin tissue of WT and *Ccr2-/-* mice re-infected with *C. auris* (n = 8-10 mice/group). (F) Percentage and absolute cell numbers (per gram tissue) of CD4 + IL17A + and CD4 + IFNγ+ (gated from TCR-β+ CD4 + cells), (G) CD4 + CD69 + IL17A + and CD4 + CD69 + IFNγ+ (gated from CD4 + CD44 + CD69 + cells) in the infected skin tissue of WT and *Ccr2-/-* mice (n = 7-12 mice/group). Error bars represent mean ± SEM. ns – non-significant, * p < 0.05, ** p < 0.01, *** p < 0.001, **** p < 0.0001. Statistical significances were calculated using the Mann-Whitney *U* test.

Next, we examined whether macrophages are a major source of IL-12 that regulates Th1 cells during *C. auris* skin reinfection. We thus examined if depletion of macrophages reduces IL-12, fungal burden, and development of Th1 cells as outlined in Fig 4D. Groups of mice were treated with either clodronate liposome (CL) or PBS on day -2 and + 2 during re-infection. As expected, CL treatment significantly decreased the percentage and absolute number of inflammatory macrophages in CL-treated mice compared to PBS-treated control groups (Fig 3E). In addition to macrophages, dendritic cells (DCs) could also be the source of IL-12. Therefore, we examined if CL treatment also alters DCs using the gating strategy as described elsewhere [25–27]. We identified monocyte-derived dendritic cells 2 (moDCs2) but not moDCs1 significantly decreased in CL-treated mice compared to PBS-treated control groups (Figs 3F and S3A). As expected, we found that IL-12 was significantly decreased in the CL-treated mice relative to PBS groups (Fig 3G). Furthermore, CL treatment significantly decreased the fungal burden in skin tissue compared with PBS-treated *C. auris*-infected mice (Fig 3H). Next, we examined the total CD4 + IL-17 + and CD4 + IFNγ+ cells in macrophage-depleted mice. We found that Th17 cells significantly increased, whereas Th1 cells significantly decreased in the CL-treated mice compared to PBS-treated control groups (Fig 3I and 3J). These findings suggest that *C. auris*-activated inflammatory macrophages and moDCs2 are the potential source of IL-12 for developing Th1 cells during secondary *C. auris* skin infection.

Next, we determined to confirm if inflammatory macrophages and moDCs regulate Th1 cells; we utilized *Ccr2-/-* mice that are defective in recruiting inflammatory macrophages and moDCs to the site of infection [28]. WT and *Ccr2-/-* mice were infected with *C. auris* to determine the fungal load, IL-12, Th1, and Th17 responses after secondary infection, as outlined in Fig 4A. We found that fungal load and IL-12p70 were significantly reduced in *C. auris*-infected *Ccr2-/-* mice compared to WT mice (Fig 4B and 4C). As expected, *Ccr2-/-* mice showed a significantly decreased number of Ly6C^high^ inflammatory macrophages and moDCs compared to WT mice during reinfection (Fig 4D and 4E). We found that Th17 cells significantly increased, whereas Th1 cells significantly decreased in the *Ccr2-/-* mice compared to WT mice groups (Fig 3F and 3G). These findings suggest that *C. auris*-activated inflammatory macrophages and moDCs are the potential source of IL-12 for developing Th1 cells during secondary *C. auris* skin infection.

### *C. auris* unique cell wall outer mannan layer regulates the development of Th1 and Th17 cells in the skin

**Fig 5:**
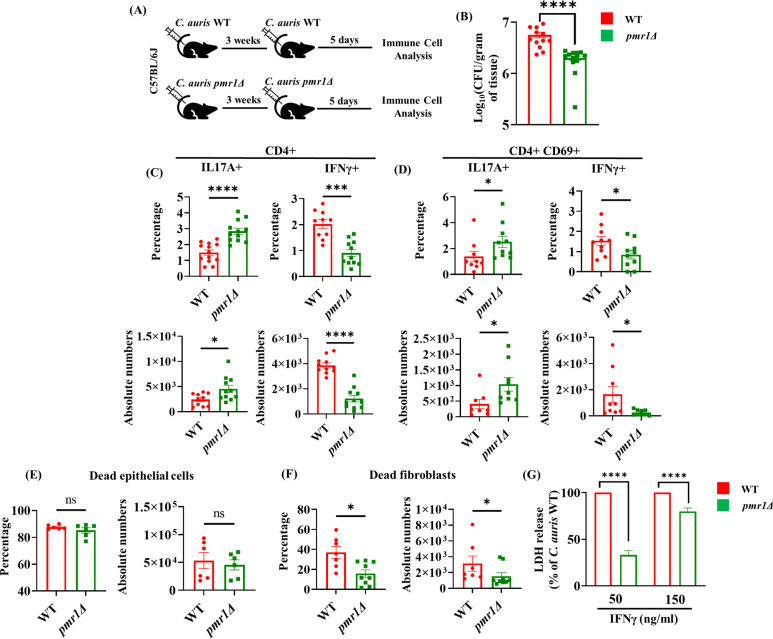
*C. auris* unique cell wall outer mannan layer regulates the development of Th1 and Th17 cells in the skin. **(A)** C57BL/6J mice were intradermally infected with WT (or) *pmr1* Δ strains of *C. auris* (dose ~ 1–2 x 10^6^/ mice) and allowed to rest for 3 weeks. Mice were re-infected with the respective WT (or) *pmr1* Δ strains of *C. auris*, and after 5 days of post-secondary infection, fungal burdens and immune cells were analyzed in the infected skin tissue. **(B)** Skin fungal burden in the *C. auris-*infected skin tissue (n = 12–15 mice per group). **(C)** Percentage and absolute cell numbers (per gram tissue) of CD4 + IL17A + and CD4 + IFNγ+ (gated from TCR-β+ CD4 + cells) T cells in WT (or) *pmr1* Δ strains of *C. auris*-infected group (n = 10–12 mice per group). **(D)** Percentage and absolute cell numbers (per gram tissue) of CD4 + CD69 + IL17A + and CD4 + CD69 + IFNγ+ (gated from CD4 + CD4 + CD69 + cells) T cells in WT (or) *pmr1* Δ strains of *C. auris*-infected group (n = 9–12 mice per group). **(E-F)** Percentage and absolute cell numbers (per gram tissue) of dead CD45^-^ CD31^-^ Tie2^-^ EpCAM^+^ epithelial cells and CD45^-^ CD31^-^ Tie2^-^ EpCAM^-^ PDGFRα^+^ fibroblasts in the skin tissue of *C. auris*-infected WT mice determined 5 days post-secondary infection (n = 6–9 mice per group). **(G)** LDH release after 24 hours of infection with WT (or) *pmr1* Δ strain of *C. auris* in mouse primary fibroblasts exposed to indicated concentrations of IFNγ (ng/ml). LDH release for *pmr1* Δ strain was calculated with respect to LDH release by primary fibroblasts infected with WT *C. auris* strain (n = 6–9 per group, data from three independent experiments combined). Error bars represent mean ± SEM. ns – non-significant, * p < 0.05, ** p < 0.01, *** p < 0.001, **** p < 0.0001. For mice studies, statistical significances were calculated using the Mann-Whitney *U* test. For *ex-vivo* LDH assays, statistical significances were calculated using the paired t-test.

Since fungal PAMPs modulate the host immune response [11], and *C. auris* possesses a unique outer mannan layer compared to *C. albicans* [12], we hypothesized that the *C. auris* outer cell wall mannan layer regulates the development of Th1 cells during *C. auris* reinfection. We used the CRISPR-CAS9 system to generate a *C. auris* mutant strain *(pmr1*Δ*)* that lacks an outer mannan layer, as described previously [29]. Wild-type B6 mice were intradermally infected with WT or *(pmr1*Δ*)* strains of *C. auris* and rested for 3 weeks. Mice were reinfected with respective strains of *C. auris* to determine the fungal load in the skin tissue (Fig 5A). We found that fungal load was significantly reduced in mice infected with *(pmr1*Δ*)* strain compared to mice infected with the WT strain of *C. auris* (Fig 5B).

Next, we examined if the unique mannan outer layer regulates the development of Th1 and Th17 cells during secondary infection. We observed that the percentage and absolute cell numbers of total CD4 + IL17A+ cells and activated CD4 + CD69 + IL17A+ cells were significantly increased, whereas total CD4 + IFNγ+ cells and activated CD4 + CD69 + IFNγ+ cells were decreased in mice infected with *(pmr1*Δ*)* strain compared with mice infected with WT strain that has intact outer mannan layer (Fig 5C and 5D). These results suggest that in the absence of a fungal outer mannan layer, *C. auris* induces a more potent Th17 response. The potent Th17 responses induced by *(pmr1*Δ*)* strain correlate with the decreased fungal load in the skin tissue.

Next, we determined to examine if the *C. auris* outer cell wall mannan layer regulates skin epithelial and fibroblast cell death during *C. auris* infection. We observed dead fibroblasts, but not dead epithelial cells, were significantly decreased in WT mice infected with the *(pmr1*Δ*)* strain compared to WT mice infected with the WT *C. auris* strain (Fig 5E and 5F). Furthermore, WT fibroblasts treated with IFNγ and infected with the *(pmr1*Δ*)* strain led to significantly decreased LDH release compared to those infected with the WT *C. auris* strain (Fig 5G). These findings suggest that *C. auris* outer cell wall mannan layer drives Th1 and Th17 responses and fibroblast cell death during *C. auris* reinfection.

## Discussion

**Fig 6:**
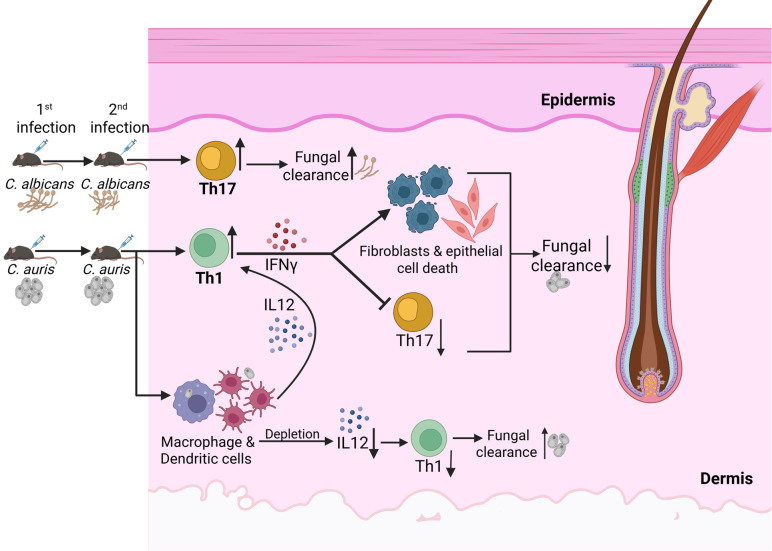
Mechanistic outline of how *C. auris* but not *C. albicans* induces IFN γ to colonize and persist in the skin (Figure created using BioRender).

Effector T cells play a critical role in the host’s defense during reinfection with *C. albicans*, *Staphylococcus aureus, Leishmania major,* and HSV [28,30–35]. Th17 cells play a critical role in host defense against *C. albicans* [30–33,36], whereas Th1 cells protect the host against reinfection from HSV and *L. major* [34,35]. However, the role of T cell immune responses during secondary infection with *C. auris,* an emerging fungal pathogen that colonizes human and mouse skin long-term, is unknown. In this study, we found that *C. auris* predominately induces potent Th1 cells in the skin after re-infection. The Th1 cytokine IFNγ enhances skin infection of *C. auris* but not *C. albicans.* The mechanism appears to be excess IFNγ dampening protective IL-17 responses and increasing barrier damage in the skin during reinfection (Fig 6). IL-17 and dermal fibroblasts play a critical role in skin defense during *C. auris* infection [6,16]. One of the potential mechanisms by which IL-17 clears pathogens is by inciting neutrophil influx. Though we observed that the recruitment of neutrophils is significantly different between *C. albicans* and *C. auris* groups, we did not notice significant differences between CL treatment and *Ccr2-/-* mice models (S3B and S3D Fig). These findings suggest that IFNγ-induced barrier damage but not neutrophils potentially contribute to increased fungal burden during *C. auris* reinfection. Previous findings from the Lionakis lab suggest that excess IFN-γ responses can impair the oral mucosal barrier and drive oral *C. albicans* infection during AIRE deficiency [21]. Our findings suggest that *C. auris* but not *C. albicans* induces IFN-γ responses, leading to barrier damage and increased fungal burden during reinfection. In addition, our findings suggest that a unique mannan layer of *C. auris* contributes to developing Th1 and Th17 cells in skin tissue.

In our model, we identified IFNγ (or) IL-17 secreting total CD4 + T cells and CD69 + activated CD4 + T cells that regulate fungal infection during *C. auris* reinfection. CD69 is an activation marker expressed by both effector T cells and tissue-resident (TRM) memory cells [37]. Previous findings suggest that IL-17-producing TRM cells play a critical role in host defense against *C. albicans* during reinfection [30–33,36]. The observed differences in Th1 and Th17 cells during *C. auris* reinfection may be due to recruited effector T cells and (or) TRM cells in the skin. However, future studies are required to understand the role of TRM versus recruited effector T cells in regulating *C. auris* during reinfection. In addition, using CL treatment in WT mice and *Ccr2-/-* mice, we identified that both inflammatory macrophages and moDCs could be a potential source of IL-12 for developing Th1 cells during reinfection. Previous findings suggest that IL-12 produced by macrophages modulates the Th1 immune response during re-infection with *L. monocytogenes* [24]. Although our current model with CL treatment in WT mice and *Ccr2-/-* mice, we found both inflammatory macrophages and moDCs could be a potential source of IL-12, future studies are necessary to dissect if inflammatory macrophages and (or) dendritic cell types that are critical for developing Th1 cells during *C. auris* reinfection in the skin. Furthermore, previous findings from our laboratory [16] and others [35] suggest that other cell types, such as keratinocytes and NK cells, also express IL-17A and IFNγ, respectively. Understanding the role of non-immune cells and NK cells during *C. auris* infection is also critical to dissect the contribution of different host factors that regulate the pathogenesis of this emerging fungal pathogen.

*C. auris* is an emerging fungal pathogen that can cause serious human infections [1,5,38,39]. *C. auris* colonizes the human skin long-term, leading to nosocomial transmission and outbreaks of skin systemic infection [6,7]. Previous findings from our laboratory and others suggest that fungal load in the skin tissue of *C. auris*-infected mice was higher than that of *C. albicans*-infected mice [6,40]. Furthermore, *C. auris* persists in mice skin tissue for over 4 months [6]. Our findings provide a new model for how *C. auris* reinfections generate Th1 cells in the skin that enhance fungal persistence. Multiple strategies have been applied to antifungal vaccine development. However, no fungal vaccine has been approved for humans [17,41]. Recent evidence suggests that NDV-3A (a vaccine based on the N-terminus of Als3 protein formulated with alum) protects mice from disseminated *C. auris* infection, although its role in protecting against skin *C. auris* infection remains unclear [42]. Taking this into account, new approaches to developing a vaccine will need to consider the generation of effector T cells and (or) TRM cells during reinfection [42], which may be the missing link in the quest for a successful vaccine against *C. auris* infections.

In this study, we used the genome-sequenced South Asian clade CA 0387 (B8441) to compare with *C. albicans* [6,43–45]. Previous findings suggest that different clades of *C. auris* elicit different innate immune responses [12]. However, previous findings from our laboratory indicated no significant differences in IL-17-mediated immune response among four clades of *C. auris* [40]. Based on these findings, we predict that effector CD4 + T cell responses may not differ among various clades of *C. auris* during reinfection. Taking together, our findings, for the first time, defined the CD4 + T cell responses to *C. auris* reinfection in the skin. Furthermore, our findings revealed a mechanism by which *C. auris* colonizes skin by inducing IFN-secreting Th1 cells, dampening protective IL-17 responses, and causing dermal damage. Future studies to understand the pathogen and host-associated molecular mechanisms governing Th1 and Th17 responses during secondary infection of *C. auris* in the skin will open the door to developing novel therapeutic approaches to prevent and treat this emerging fungal pathogen.

## Methods

### Ethics statement

Mice were maintained under pathogen-free conditions. All animal studies were performed, and experimental protocols were approved by the Purdue University Institutional Animal Care and Use Committee (IACUC Protocol no. 2110002211).

### Materials and reagents

The following reagents used in the study – YPD (BD Biosciences, Cat# 242810), agar (Thermofisher, Cat# BP1423–500), 10X PBS (Thermofisher, Cat# BP3394), ampicillin (Cayman Chemicals, Cat# 14417), Streptomycin (MP Biomedicals, Cat# 100556), DMEM (Gibco, Cat# 11-965-092), FBS (CPS Serum, FBS-500HI), Triton X-100 (MP Biomedicals, Cat# 807423), RPMI 1640 (Thermofisher. Cat# 11875093), Eagle’s Minimum Essential Medium (ATCC, Cat# 30–2003), DMEM/High glucose with L-glutamine, sodium pyruvate (Cytiva, Cat# SH30243.02), Trypsin-EDTA (0.25%) (Thermofisher, Cat# 25200056), keratinocyte growth medium (Sigma Aldrich, Cat# C-20111), dispase II (Sigma Aldrich, Cat# D4693), antibiotic-antimycotic solution (Sigma Aldrich, Cat#A5955), SYBR Green master mix (Applied Biosystems, Cat# A25742), clodrosome (Encapsula Nano Sciences, Cat# CLD-8909), anti-IL17A (BioXCell, Cat# BE0173), liberse TL (Sigma Aldrich, Cat#05401020001), DNase I (Sigma Aldrich, Cat# DN25-100G). ELISA MAX deluxe set mouse IL-12 (p70) (Cat# 433604), LDH cytox kit (Cat# 426401), recombinant mouse IFN-γ (Cat# 575304), anti-mouse CD8a (Cat# 100763), cell staining buffer (Cat# 420201), intracellular staining permeabilization wash buffer (Cat#421002), fixation buffer (Cat# 420801), cell activation cocktail (without Brefeldin) (Cat# 423302) was purchased from Biolegend. 3T3-L1 (Cat# CL-173) mouse fibroblast cell line was purchased from ATCC. Kera-308 (Cat# 400429) mouse keratinocyte cell line was purchased from Cytion. The flow cytometry antibodies were purchased from Biolegend - CD11c (Cat# 117309), Ly6C (Cat# 128014), CD11b (Cat# 101208), Ly6G (Cat# 127618), CD45 (Cat# 103108), CD64 (Cat# 139320), TCR γ/δ (Cat# 118120), CD4 (Cat# 100434), TCRβ (Cat# 109222), CD44 (Cat# 103008), CD69 (Cat# 104514), CD31 (Cat# 102422), CD45 (Cat# 103126), CD140a (Cat# 135912), CD202b (Cat# 124008), CD326 (Cat# 118240), IL17A (Cat# 506938), IFN-γ (Cat# 505824), CD16/32 (Cat# 101302). LIVE/DEAD fixable yellow (Cat# L34959) and MHC-II (Cat# 56-5321-82) were purchased from Thermofisher Scientific.

### Mice

The C57BL/6J (WT), B6.129S7-*Ifng*^*tm1Ts*^/J (*Ifng-/-*) (Strain #: 002287), and B6.129S4-*Ccr2*^*tm1Ifc*^/J (*Ccr2-/-*) (Strain #: 004999) mice were bred and housed at the centrally managed animal facility in Purdue University. All mice were purchased from Jackson Laboratory. All experiments used age- and sex-matched mice between 6 and 12 weeks old.

### Fungal strains

The strains used in the study were *Candida albicans* SC5314 (obtained from Dr. Andrew Koh, University of Texas Southwestern Medical Center, USA) and *Candida auris* AR0387 (obtained from CDC AR Isolate Bank, USA). *Candida auris* AR0387 *(pmr1*Δ*)* was created in the lab as described previously. [16]. The stock culture of the fungal stains was stored at - 80°C and was streaked on yeast peptone dextrose (YPD) agar plates. The YPD agar plates were incubated at 30°C for 24 hours (hr).

### Murine skin infection

 For murine skin infection, fungal strains were incubated in YPD media overnight with 250 rpm shaking. Cells were washed twice with sterile 1 × PBS and counted to 1–2 x 10^7^ yeast cells/ml under a hemocytometer. Mice were anesthetized, and dorsal skin hair was trimmed and removed as previously described [40]. Mice were injected intradermally with 1–2 x 10^6^ yeast cells on the shaved right flank of the dorsal part using a 27 G 1’ hypodermic needle. Mice were rested for 3 weeks. During secondary infection after 3 weeks, mice were injected intradermally with ~ 1–2 x 10^6^ yeast cells on the shaved left flank (opposite to the previous injection site) of the dorsal part. Each figure and figure legend indicate the details of each experiment (study outline, sample size).

### Macrophage depletion in mice

 To deplete macrophages during secondary infection (3 weeks after primary infection), mice were injected subcutaneously with 1 mg of clodrosome (Encapsula Nano Sciences) on day -2 and + 2 following *Candida* skin infection. 1 × PBS was injected into the control groups. Depletion was confirmed by flow cytometry.

### Antibody administration

 For neutralizing experiments, *Ifng-/-* mice were administered with anti-IL17A (BioXCell, clone 17F3) intraperitoneally at a dose of 500 μg per mouse on days -1, + 1, + 3 following *Candida* skin infection. The isotype control antibody with the same dose was administered to the control group. For CD8 T cell depletion, C57BL/6J mice were administered with anti-mouse CD8a (Biolegend, clone 53-6.7) intraperitoneally at a dose of 400 μg per mouse on days -2, + 2 following *Candida* skin infection. Fungal burden in skin tissues was determined on day 5 of post-secondary infection.

### Fungal burden determination in skin tissue

 After 5 days of secondary infection, mice were euthanized, and the infected skin tissues were collected and weighed. The tissues were homogenized using 1 × PBS, and the homogenates were serially diluted and plated in antibiotic-containing YPD-agar plates. The plates were incubated overnight at 30°C to determine the fungal burden in the skin tissues.

### Skin digestion and flow cytometry

 After 5 days post-infection (or stated otherwise), mice were euthanized, and skin tissues were collected. To check the cell death of non-immune cells like fibroblasts and epithelial cells, skin samples were collected adjacent to the secondary infection site (non-inclusion of injection wound site). Single-cell suspensions were prepared as described previously. [27,40]. Briefly, the excised skin was minced using ophthalmic scissors and was incubated in digestion media (RPMI-1640 with 0.25-mg/mL Liberase TL and 1-µg/mL DNase) in a CO_2_ incubator for 2 hours at 37°C. Before the end of the digestion step, 0.25% trypsin-1-mM EDTA was added to separate the dermis from the epidermal surface. The tissues were then pumped 8–10 times with a 10-mL syringe to dissociate the release of single cells from the tissues mechanically. Cells were filtered through a 40 μm cell strainer centrifuged at 400 x g for 10 min and resuspended in chilled PBS with 5% FBS. Cells were transferred to a U-bottom 96-well plate for the staining procedure. The single-cell suspensions were stained with LIVE/DEAD Fixable Yellow (Invitrogen, Waltham, MA, USA), followed by surface markers for defining innate immune and non-immune cells. For adaptive T cell staining, mononuclear cells were stimulated with a cell activation cocktail without brefeldin (1:500) and monensin (1:1,000) for 4.5 hours at 37°C at 5% CO_2._ Cells were stained with LIVE/DEAD Fixable Yellow, followed by surface and intracellular markers for defining adaptive T cell populations. The stained samples were acquired through Attune NxT Flow Cytometer (Invitrogen, Carlsbad, CA, USA) and analyzed using FlowJo software (Eugene, OR, USA).

### Preparation of single-cell suspension from murine skin tissue and single-cell RNA sequencing.

For scRNA seq, mice were injected intradermally with either ~ 1–2 x 10^6^ yeast cells (*C. auris* infected group) or sterile 1 × PBS (uninfected group) on the shaved dorsal skin area. After 12 days of post-infection, mice were euthanized, and single-cell suspension was prepared, as described previously [16]. The viability of the cells was measured under a microscope, and samples with more than 85% viability and less cell debris were enumerated and sent for single-cell partition and RNA library preparation. The single-cell suspensions were loaded on a microfluidics chip for further processing. cDNA was synthesized following cell capture and cell lysis, and the final libraries were generated using Illumina NovaSeq 6000. The FASTQ files were generated using CellRanger (v7.1.0) pipeline from 10X Genomics. Differential gene expression between uninfected and infected samples was performed using edgeR [46]. Heatmaps were generated using Single R package [47]. Application of statistical models to identify the genes that were significantly differentially expressed were generated, based on FDR ≤ 0.05 and Log2FC ≥ 2 or ≤ -2 between infected and uninfected conditions for each cell type as described previously [16].

### RNA isolation and quantitative RT-PCR

 Mouse skin tissues were collected in TRIzol after euthanasia and were kept at -80°C. Tissues were homogenized in TRIzol, and the mRNA was extracted from skin tissues using TRIzol as per the manufacturer’s recommendation. mRNA was then converted to cDNA using a high-capacity cDNA Reverse Transcription Kit (Applied Biosystems). Quantitative real-time PCR (qPCR) was performed with SYBR Green FastMix. The following genes were analyzed: *Il6, Il1b, Cxcl1,* and *S100a8*. The expression of each gene was normalized to that of beta-actin by calculating 2^−ΔCt^. The primer for the genes was purchased from Integrated DNA Technologies; *Il6* (F: 5’TACCACTTCACAAGTCGGAGGC3’, R: 3’CTGCAAGTGCATCATCGTTGTTC5’), *S100a8* (F: 5’CAAGGAAATCACCATGCCCTCTA3’, R: 3’ACCATCGCAAGGAACTCCTCGA5’), *Il1b* (F: 5’TGGACCTTCCAGGATGAGGACA3’, R: 3’GTTCATCTCGGAGCCTGTAGTG5’), *Cxcl1* (F: 5’TCCAGAGCTTGAAGGTGTTGCC3’, R: 3’AACCAAGGGAGCTTCAGGGTCA5’).

### ELISA

 The skin homogenates were centrifuged at 4000 rpm for 10 min at 4^0^C, and supernatants were stored at – 20°C. 100 µl of supernatants from the skin homogenate were used for measuring IL12 level in the mouse skin using an ELISA MAX Deluxe Set Mouse IL-12 (p70) (BioLegend) by following the manufacturer’s standard instructions.

### Fibroblast isolation and culture.

Mouse dermal fibroblast was isolated from the underarm area of C57BL/6J mice following the protocol described here [48]. Briefly, the underarm areas of 6–8 weeks C57BL/6J mice after euthanasia were soaked with 70% ethanol, and then the fur was removed using chemical depilatory cream. Approximately 1 cm^2^ skin fragments were collected and kept in 50ml tubes containing sterile PBS to avoid drying. The skin tissue was then cut into ~1 mm pieces using two sterile scalpels and subjected to tissue digestion in 0.14 wunch units per ml of Liberase containing DMEM media with 1 × antibiotics/ antimycotic solution for 1- 1.5 hours at 37°C in a beaker with gentle stirring. After completion of skin digestion, once the digestion media became turbid, digestion was stopped using DMEM media with 15% FBS and 1 × antibiotics/antimycotic solution (complete DMEM media), and digested tissue fragments were pipetted vigorously to break the clumps. Subsequently, the tissue fragments were cultured in complete DMEM media for 14 days with intermittent media change after 7 days post-isolation to facilitate the exit of fibroblasts from tissue fragments. After 14 days, once the culture flask attained confluency, the tissue fragments were discarded, and the cells were switched to complete EMEM media to promote the growth of only fibroblast cells and to stop the proliferation of other cell types. The cells selected in EMEM media were then sub cultured as needed and used in subsequent experiments.

### Assessment of viability in mouse primary fibroblasts

 Mouse primary fibroblasts were seeded at 5x10^3^ cells per well in 96 well plates, and after 2 days, cells were stimulated with complete DMEM containing 50 ng/ml and 150 ng/ml of IFN-γ for 24 hours. All experiments were conducted at > 90% confluency. The extent of cell damage in fibroblasts caused by IFN-γ in the absence or presence of WT (or) *(pmr1*Δ*)* strain of *C. auris* was measured using (Lactate dehydrogenase) LDH release assay. Cell death of primary cells using LDH release assay was calculated using this calculation: 1. $Extent\;ofcelldeath=\frac{\left[(SampleLDHrelease)-(ControlLDHrelease)\right]}{\left[(TotalLDHrelease)-(ControlLDHrelease)\right]}*100.$ Briefly, fibroblasts were incubated with 50 ng/ml and 150 ng/ml of IFN-γ for 24 hours, and stimulated fibroblasts were infected with WT (or) *pmr1* Δ strains of *C. auris* at an M.O.I 1:2. Uninfected fibroblasts cells were processed in parallel and were used as control. Triton-X was used to determine the total LDH release by the primary cells. After 24 hours of infection, LDH release in the supernatant was quantified using the LDH-Cytox Assay Kit (BioLegend) as recommended by the manufacturer. Each experiment was performed three independent times.

### Keratinocyte isolation and culture.

Mouse epidermal keratinocytes were isolated from the tail skin of C57BL/6J mice following the protocol described previously [49]. Briefly, 6–8 weeks C57BL/6J mice were euthanized, and the tail was aseptically excised by soaking with 70% ethanol. Subsequently, the tail skin was peeled off using two forceps, and the peeled skin was rinsed with sterile PBS before placing it in ice-cold dispase digestion buffer (4mg/ml dispase in keratinocyte growth medium) overnight at 4°C refrigerator on a rotator. The next day, the skin tissue was subjected to the separation of the epidermis from the dermis layer, and the separated epidermis was floated on 0.25% Trypsin EDTA solution with the basal layer downward for 20 minutes. The basal layer digestion ended using the appropriate keratinocyte growth medium, and the epidermal sheet was then rubbed vigorously back and forth to release the single cells. The collected cell suspension was then gently pipetted to break any cell clump, passed through a 100 µ M filter, and centrifuged to settle the cells. The cell pellet was then resuspended and cultured in a keratinocyte growth medium for 5 days and then used for further experiments.

### Assessment of viability in mouse primary keratinocytes

 Mouse primary keratinocytes were seeded 5x10^4^ cells per well in 48 well plates. After 2 days, cells were stimulated with a keratinocyte medium containing 50 ng/ml and 150 ng/ml of IFN-γ for 24 hours. All experiments were conducted at > 90% confluency. The extent of cell damage in keratinocytes caused by IFN-γ in the absence or presence of WT (or) *pmr1* Δ strain of *C. auris* was measured using LDH release assay. Briefly, keratinocytes were incubated with 50 ng/ml and 150 ng/ml of IFN-γ for 24 hours, and stimulated keratinocytes were infected with WT (or) *pmr1* Δ strain of *C. auris* at a multiplicity of infection (MOI) of 1:2. Uninfected keratinocytes was used as control. After 24 hours of infection, LDH release in the supernatant was quantified. Each experiment was performed three independent times.

### Determination of fungal burden in keratinocytes and fibroblasts cell line

 The mouse 3T3-L1 (ATCC) fibroblast cell line and mouse skin Kera-308 keratinocyte cell line (Cytion) were used for determining the *C. auris* survival and proliferation assay in the presence of IFNγ. The cells were grown in cDMEM medium for 3–4 days and then were used for experiments. The mouse skin Kera-308 keratinocytes were seeded 2x10^4^ cells per well in 96-well tissue culture plates. After 2 days, cells were stimulated with cDMEM containing 150 ng/ml of IFNγ for 24 hours. Similarly, the mouse 3T3-L1 fibroblasts were seeded 2x10^4^ cells per well in 96-well tissue culture plates. After 1 day, fibroblasts were stimulated with cDMEM containing 150 ng/ml of IFNγ for 24 hours. All experiments were conducted at > 90% confluency. After 24 hours of stimulation, both fibroblasts and keratinocytes with > 90% confluency were infected with *C. auris* 0387 at an MOI of 1:1. Unstimulated cells were infected with the same M.O.I. in parallel and were used as a control. After 24 hours of infection, *C. auris* survival was quantified by plating the fungi in antibiotic-containing YPD-agar plates. The plates were incubated overnight at 30°C to determine the fungal survival and proliferation in the presence or absence of IFNγ in ex-vivo fibroblasts or keratinocytes culture.

### Statistical analysis

 Statistical analysis was performed using the Mann-Whitney *U* test for all mice studies. Individual data points in each bar graph represent each mouse for all mice studies. For ex-vivo experiments, statistical analysis was performed using the paired t-test using GraphPad Prism 9.4.1 (GraphPad Software, La Jolla, CA). *P* values of ≤ 0.05 were considered significant.

## Supporting information

S1 Fig (A) Flow cytometry gating strategy of γδ + T cells, CD4 + T and CD4 + CD44 + CD69 + T cells in the murine skin. **(B)** Representative flow plots of CD4 + IL17A + and CD4 + IFNγ+ (gated from TCR-β+ CD4 + cells) T cells in the uninfected, *C. albicans* and *C. auris*-infected mice skin after 5 days of re-infection. **(C)** Fungal burden in the skin of *C. auris*-infected WT mice after 5 days post-secondary infection that receives anti-CD8a or isotype antibody (n = 5 mice per group). Error bars represent mean ± SEM. ns - non-significant. Statistical significances were calculated using the Mann-Whitney *U* test.
(TIF)

S2 Fig (A) Flow cytometry gating strategy of skin epithelial and fibroblast cell populations. CD45^-^ CD31^-^ Tie2^-^ EpCAM^-^ PDGFRα^+^ fibroblasts and CD45^-^ CD31^-^ Tie2^-^ EpCAM^+^ epithelial cells are shown here. **(B)** Fungal burden was assessed after 24 hours of *C. auris* infection in the Kera-308 keratinocyte cell line in the presence or absence of IFNγ (150 ng/ml). (combined data from four independent experiments). **(C)** Fungal burden was assessed after 24 hours of *C. auris* infection in the 3T3-L1 fibroblast cell line in the presence or absence of IFNγ (150 ng/ml). (combined data from four independent experiments). Error bars represent mean ± SEM. * p < 0.05, ** p < 0.01. Statistical significances were calculated using the paired t-test.
(TIF)

S3 Fig (A) Flow gating strategy for neutrophils, Ly6C^high^ inflammatory macrophages (Ly6C^high^ MHC-II +), and monocyte-derived dendritic cells (moDC 1 and moDC2) in mouse skin. **(B)** Percentage and absolute numbers per gram of tissue of CD11b + Ly6G+ neutrophils among *C. albicans* and *C. auris*-infected mice after 5 days of post-secondary infection (n = 10–13 mice/group). **(C)** Percentage and absolute numbers per gram of tissue of CD11b + Ly6G+ neutrophils among control and macrophage-depleted mice after 5 days of post-secondary infection with *C. auris* (n = 9–10 mice/group). **(D)** Percentage and absolute numbers/gram of tissue of CD11b + Ly6G+ neutrophils among WT and *Ccr2-/-* mice after 5 days of post-secondary infection with *C. auris* (n = 9–10 mice/group). Error bars represent mean ± SEM. ns - non-significant, * p < 0.05, **** p < 0.0001. Statistical significances were calculated using the Mann-Whitney *U* test.
(TIF)
