# Supplementary figures and images for: The Emerging Fungal Pathogen Candida auris Induces IFNγ to Colonize the Skin
Source: PLoS Pathog. 2025 Apr 28;21(4):e1013114. doi: 10.1371/journal.ppat.1013114 (PMC12121905; doi:10.1371/journal.ppat.1013114)

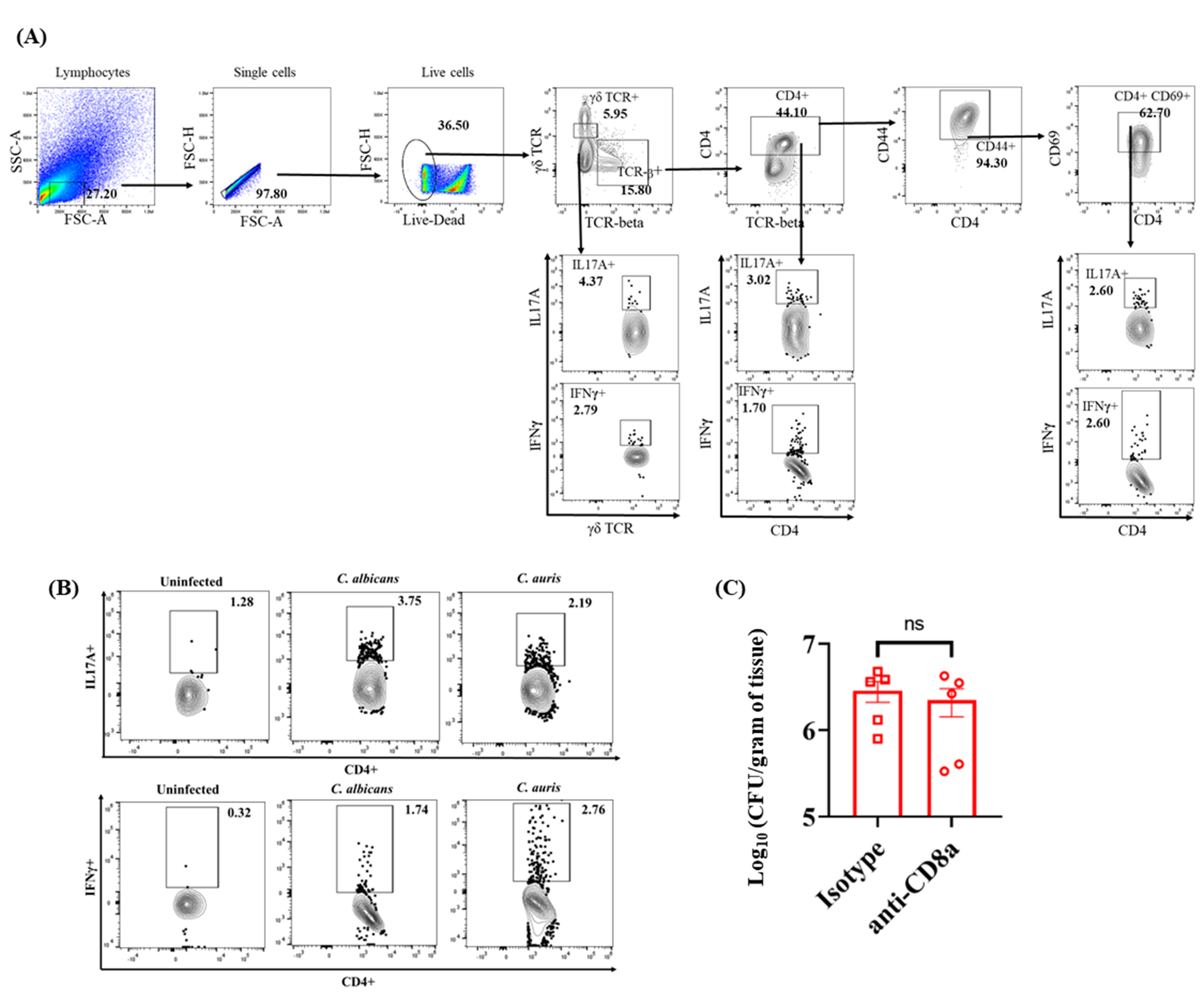

Supplement: S1 Fig — (B) Representative flow plots of CD4 + IL17A + and CD4 + IFNγ+ (gated from TCR-β+ CD4 + cells) T cells in the uninfected, C. albicans and C. auris-infected mice skin after 5 days of re-infection. (C) Fungal burden in the skin of C. auris-infected WT mice after 5 days post-secondary infection that receives anti-CD8a or isotype antibody (n = 5 mice per group). Error bars represent mean ± SEM. ns - non-significant. Statistical significances were calculated using the Mann-Whitney U test. (TIF) [file ppat.1013114.s001.tif]

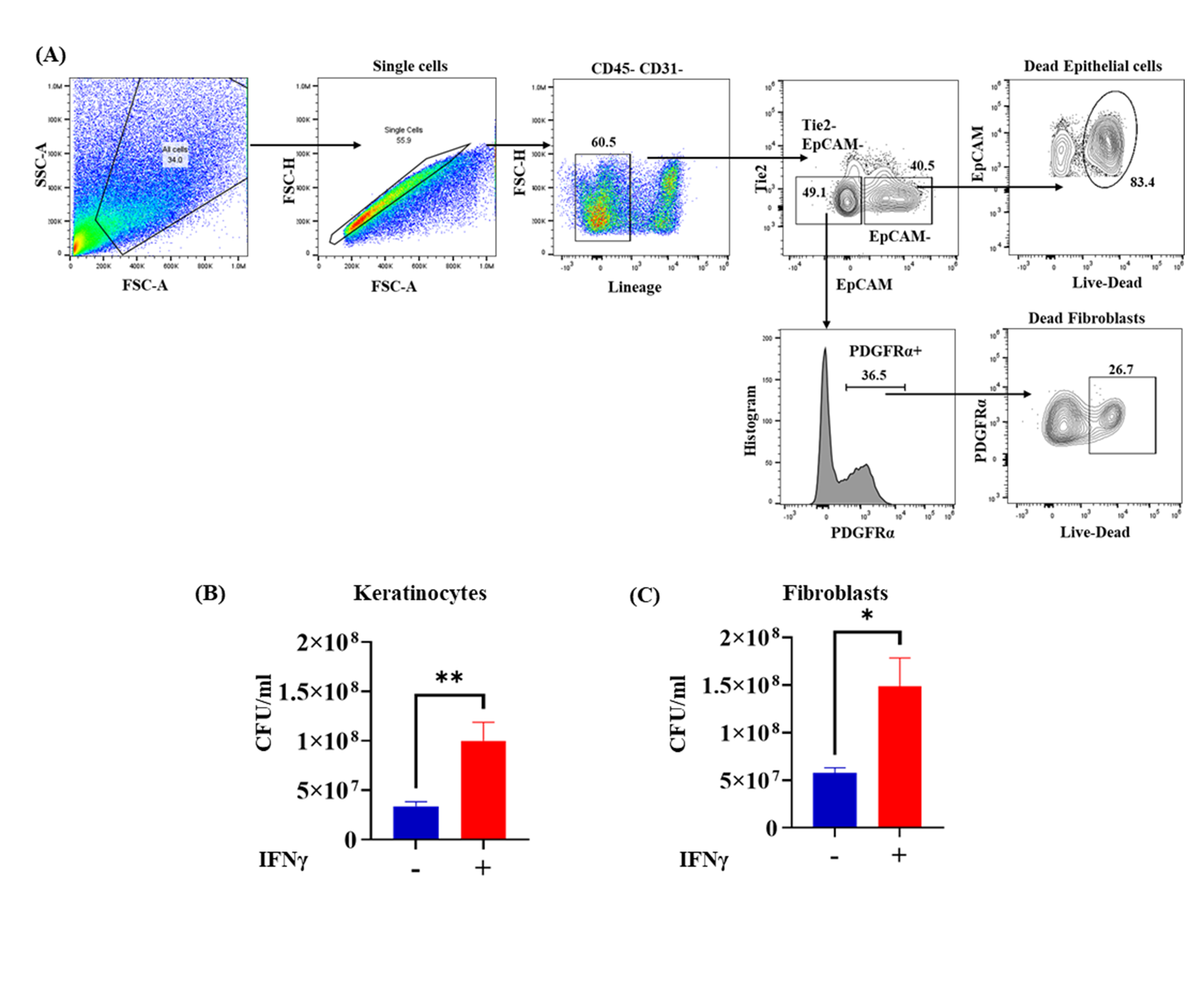

Supplement: S2 Fig — CD45- CD31- Tie2- EpCAM- PDGFRα+ fibroblasts and CD45- CD31- Tie2- EpCAM+ epithelial cells are shown here. (B) Fungal burden was assessed after 24 hours of C. auris infection in the Kera-308 keratinocyte cell line in the presence or absence of IFNγ (150 ng/ml). (combined data from four independent experiments). (C) Fungal burden was assessed after 24 hours of C. auris infection in the 3T3-L1 fibroblast cell line in the presence or absence of IFNγ (150 ng/ml). (combined data from four independent experiments). Error bars represent mean ± SEM. * p < 0.05, ** p < 0.01. Statistical significances were calculated using the paired t-test. (TIF) [file ppat.1013114.s002.tif]

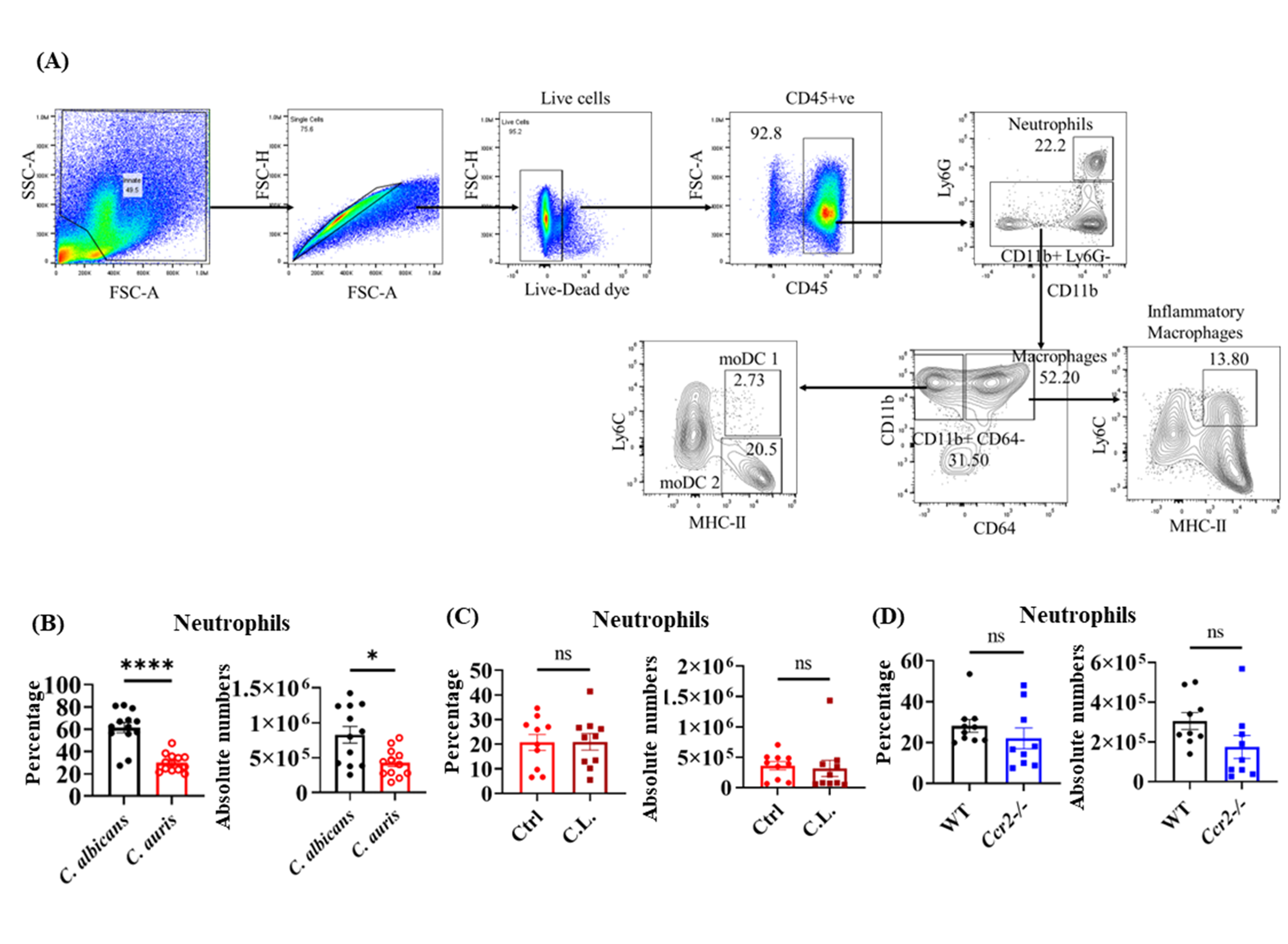

Supplement: S3 Fig — (B) Percentage and absolute numbers per gram of tissue of CD11b + Ly6G+ neutrophils among C. albicans and C. auris-infected mice after 5 days of post-secondary infection (n = 10–13 mice/group). (C) Percentage and absolute numbers per gram of tissue of CD11b + Ly6G+ neutrophils among control and macrophage-depleted mice after 5 days of post-secondary infection with C. auris (n = 9–10 mice/group). (D) Percentage and absolute numbers/gram of tissue of CD11b + Ly6G+ neutrophils among WT and Ccr2-/- mice after 5 days of post-secondary infection with C. auris (n = 9–10 mice/group). Error bars represent mean ± SEM. ns - non-significant, * p < 0.05, **** p < 0.0001. Statistical significances were calculated using the Mann-Whitney U test. (TIF) [file ppat.1013114.s003.tif]
